# Trials and Tribulations: The ‘Use’ (and ‘Misuse’) of Evidence in Public Policy

**DOI:** 10.1111/spol.12024

**Published:** 2013-06-07

**Authors:** Christopher Deeming

**Affiliations:** Geographical Sciences, University of BristolBristol, UK

**Keywords:** Social research, Evidence-based policy, International comparisons, Knowledge utilization, Impact evaluation

## Abstract

Randomized controlled trials (RCTs) are increasingly playing a central role in shaping policy for development. By comparison, social experimentation has not driven the great transformation of welfare within the developed world. This introduces a range of issues for those interested in the nature of research evidence for making policy. In this article we will seek a greater understanding of why the RCT is increasingly seen as the ‘gold standard’ for policy experiments in low- and middle-income countries (LMICs), but not in the more advanced liberal democracies, and we will explore the implications of this. One objection to the use of RCTs, however can be cost, but implementing policies and programmes without good evidence or a good understanding of their effectiveness is unlikely to be a good use of resources either. Other issues arise. Trials are often complex to run and ethical concerns often arise in social ‘experiments’ with human subjects. However, rolling out untested policies may also be morally objectionable. This article sheds new light on the relationship between evidence and evaluation in public policy in both the global north and developing south. It also tackles emerging issues concerning the ‘use’ and ‘misuse’ of evidence and evaluation within public policy.

## Introduction

We can achieve a sort of control under which the controlled, though they are following a code much more scrupulously than was ever the case under the old system, nevertheless feel free. They are doing what they want to do, not what they are forced to do. That's the source of the tremendous power of positive reinforcement – there's no restraint and no revolt. By careful cultural design, we control not the final behaviour, but the inclination to behave – the motives, desires, the wishes. (Skinner 1948: 246–7)

Many observers have commented on the spread of behavioural experiments in global policy. This new and emerging form of public policy often involves conditioning the receipt of welfare – in the form of cash transfers, goods or services – according to specific individual behaviour. Few however, have touched on the contextual differences in terms of the application of research and evidence governing policy, especially social assistance in the developing world and social security within the developed world. Subsequently, the debates on the progress of public policy in the global north and south are not as well connected as they might be.

In LMICs, social experiments with conditionality are part of the drive for evidence-based policy (Fiszbein *et al*. 2009). Conditional cash transfer (CCT) programmes usually have an *a priori* evaluation design built into their operation, that embraces experimental or quasi-experimental features and RCT designs, for example. By contrast, in the advanced liberal democracies, there has been less direct appeal to research evidence gained using from robust evaluation in order to secure major welfare reform: experimentation and evaluation with RCTs has been less of a priority. Consequently, welfare states were reformed on ideological grounds, with an appeal to political theory as Mead and Beem (2005) observe. Putting this in stark terms, welfare conditionality in the south is, arguably, being driven by an evidence-based policy-making agenda, whereas in the north, political philosophy is clearly driving welfare reform. This article seeks to shed new light on the relationship between evidence and evaluation within the different worldly contexts, by drawing out emerging arguments and counter-arguments about the ‘use’ and ‘misuse’ of evidence within public policy. The article is organized as follows: the first section examines the increase and nature of evidence-based policy for development; the second section considers how welfare policy has been transformed in the developed world; followed by a more detailed examination of some of the controversies in section three. Lastly, reflections and conclusions are drawn together in the fourth section.

## Public Policy for Development: The Rise of Behavioural Economics

**Figure 1:**
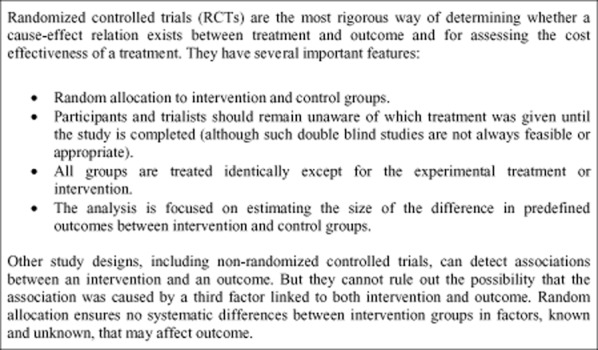
Randomized controlled trials (RCTs) *Source:* adapted from Sibbald and Roland 1998: 201.

**Figure 2:**
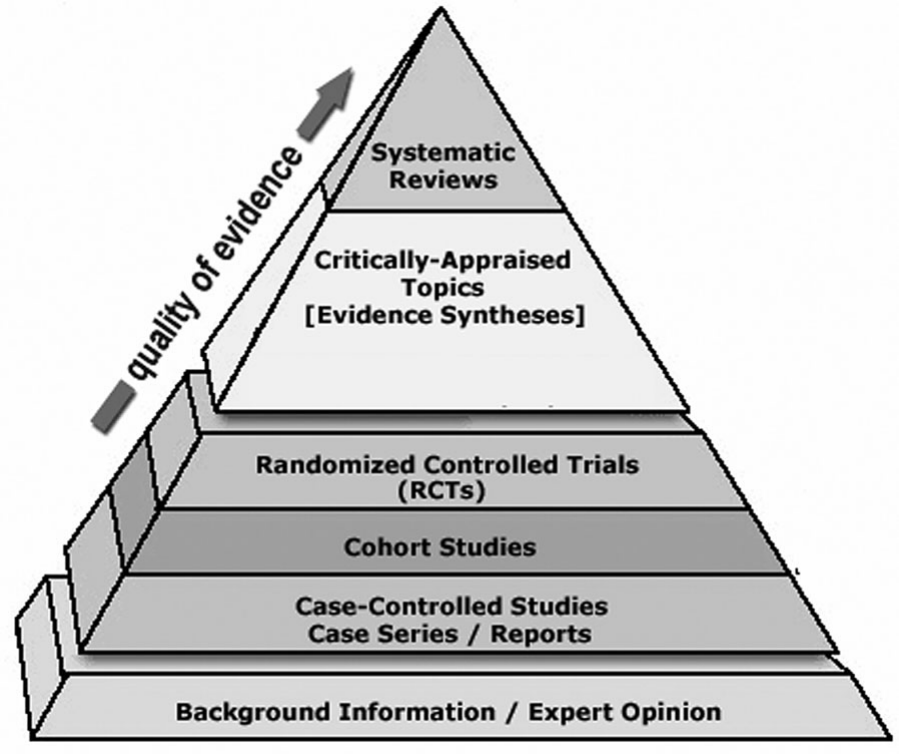
Hierarchies of research evidence *Source:* adapted from Davies *et al*. 2000: 48.

In the brave new world of the behavioural economist, achieving the Millennium Development Goals (MDGs) for health and well-being is ultimately about demonstrably changing people's behaviour for the better. Being able to demonstrate the effectiveness or impact of an intervention is thus the keystone for policy development. As a result of the global research effort, it is now well established that there are certain (desirable) human behaviour and conditions of living that are beneficial for our health and well-being that, arguably, should form the basis for public policy (Dean 2010). This point, implicit in the literature, certainly needs to be made more explicit, as biomedical and social research continues to establish food and dietary requirements for good health, education, housing and living standards, along with certain behaviour and practices that reduce the risk of ill-health and disease (e.g. WHO 2002). For far too long, the criticism has been that acceptance of incontrovertible evidence into functioning policy has been slow, partial and unsystematic; resulting in health deficits, waste of human potential and other associated costs to society (WHO 2011). The second point, about clearly demonstrating success and impact in social policy addresses the need for robust evaluation. In order to consider the effectiveness of a particular policy or programme, one really needs to know what outcomes would have been achieved had the programme not been in place. This is often referred to as the ‘counterfactual outcome’ (OECD n.d.). One way of overcoming this evaluation problem is through the use of RCTs, which are considered to be the ‘gold standard’ of all the methods available to researchers (Young *et al*. 2002). In the trials, participants are usually randomly assigned to intervention or control groups (Sibbald and Roland 1998), researchers then compare the outcomes between groups (see figure 1). A systematic review of RCT results can usually be found at the very pinnacle of the research evidence hierarchy of (figure 2), as it provides a way of pooling evidence from different studies to provide an overview of outcomes (White and Waddington 2012).^1^

**Figure 3:**
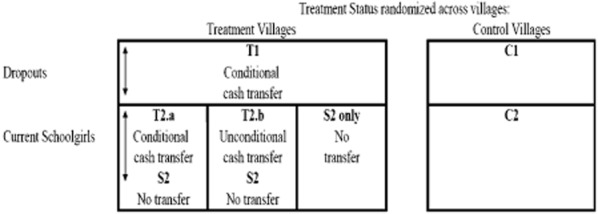
Malawi RCT design *Source:* Baird *et al*. 2009a: 7.

It may not be surprising, therefore, to find an influential group of economists advocating social experiments and in particular RCTs, as the main tool for studying the effectiveness of policy in development settings. In a recent trial in Malawi, for example, poor families were given cash on the condition that they send their children to school (Baird *et al*. 2000a, 2000b). Some villages were randomly assigned to the social assistance programme and others were not (figure 3). Importantly, at the outset, each village had the same equal chance of being picked to receive the intervention; families were not told that they were part of an experiment, they were ‘blind’ or ‘blinded’: knowing this may affect their behaviour and the outcome of the study. The development economists – sometimes referred to as the ‘randomistas’ – argue that this type of randomized experiment is the only sure way of identifying impact, because it helps to eliminate bias and other confounding (hidden) factors. Other non-experimental methods found at the bottom of the evidence hierarchy are largely dismissed as they are considered unscientific and are best avoided (figure 2). The influence of the randomistas appears to be growing; non-governmental organizations (NGOs) (the World Bank, the International Monetary Fund, the World Trade Organization), philanthropic agencies and donors (e.g. DFID 2011b) are increasingly giving (explicit) preference to randomized designs and systematic reviews in evaluating public policy programmes and their impacts (Hickey *et al*. 2009; Hagen-Zanker *et al*. 2012).

**Figure 4:**
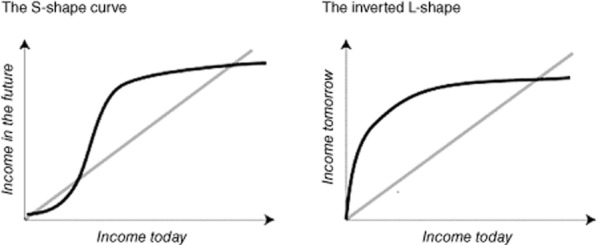
Different prospects for the world's poorest *Source:* adapted from Banerjee and Duflo 2011: 12–13.

According to behavioural economists like Banerjee and Duflo (2011), the major debates in international development can be boiled down to disagreements about the shape of a function in development theory (figure 4). The S-shaped curve on the left suggests poor people are ‘trapped’ in poverty and require a ‘conditional push’ to get out of their own poverty. The L-shaped curve on the other hand, suggests that poor people are gradually able to pull themselves out of poverty because they are not really ‘trapped’ at all. If we accept the premise of the development economist, the theoretical proposition and corresponding debate about which of the two graphs best represents the real world can only be settled experimentally, through the use of RCTs. Of course, not all social researchers agree with the world view depicted in these two charts or see the need for social experiments to solve the problem of global poverty. The randomistas, however, firmly believe that it is perfectly possible to make significant progress in tackling global social problems using experimentation; through the accumulation of small experimental steps, each well thought out, carefully tested and judiciously implemented. In effect, they claim to be saving lives with a well-placed behavioural ‘nudge’ and they offer plenty of evidence to support their position.

### Saving lives with a well-placed nudge

Conditional social policy programmes are now firmly established across the developing world and shape the lives of millions of people. Many CCT programmes are large-scale and usually have an evaluation design built into their operation. *Oportunidades* in Mexico for example, covers about a quarter of Mexico's national population (1.5 million households). In Brazil, some 11 million families – 46 million people – receive regular transfers under the *Bolsa Família* programme. Mexico's *Progresa* (renamed *Oportunidades* in 2002), often seen as the seminal and model programme, began in 1997. This programme has demonstrated a range of benefits over the years, particularly in the areas of health and education (DFID 2006). For instance, some 70 per cent of families participating in the scheme have shown improved nutritional status and stunting has been reduced; ante-natal care increased by 8 per cent, contributing to a 25 per cent drop in the incidence of illness in newborns; immunization rates improved as a result of preventive healthcare appointments; and school enrolment rates increased by over 20 per cent for girls and by 10 per cent for boys. These social assistance programmes also have fiscal appeal, at least according to national governments and NGOs such as the World Bank. In terms of national budget, Mexico and Brazil commit only 0.5 per cent of gross domestic product to their programmes. With the evidence-base for CCT programmes growing, Guatemala is one of the latest Latin American countries to embark on reform with *Mi Familia Progresa* (my family is moving forward), introduced in 2008 (Gaia 2010). In lower-income contexts, especially in Africa, CCT programmes and experiments are often on a smaller scale – as well as familiar conditions relating to school attendance and the use of health services, other conditions continue to be tested, for example adult education, micro-credit, housing and accommodation schemes, also bed net schemes to help protect people against malaria-carrying mosquitoes (DFID 2011a).

The evidence suggests CCT programmes can be particularly effective in promoting health and education services in LMICs (Lagarde *et al*. 2007). Improvements in the use of health services (table 1) is particularly striking as the effects are concentrated among families who are least likely to use the services in the absence of conditional reward. School enrolment rates also show significant gains with conditionality in place (table 2). In addition, there are demonstrable impacts on family food consumption. Table 3 shows significant increases in daily per capita food consumption: up by 12 per cent in Brazil, 6 per cent in Colombia and over 30 per cent in Nicaragua. Families in the intervention group also tend to spend significantly more on food, indicated by the findings on food budgets. For example, household food expenditures as a proportion of total household budgets increased by 4 percentage points among programme beneficiaries in Columbia, Ecuador and Nicaragua (table 3). Lastly, CCT programmes have secured significant reductions in levels of extreme poverty (table 4). In Mexico, for instance, severe poverty as measured by the ‘squared poverty gap’, which attempts to take account of the depth of poverty and inequality among poor people, has fallen by about 29 per cent. Systematic reviews continually look to refine and improve the effectiveness of CCT programmes (Gaarder *et al*. 2010; Yoong *et al*. 2012).^2^ Despite the huge investments in large-scale trials, important questions remain unanswered, particularly concerning cost-effectiveness and cost-benefit, an issue to which we return below. Before this, however, we turn to consider the transformation of social policy in the advanced economies, where concerns about the paucity of evidence and robust evaluation continue to be raised.

**Table 1:** Impact of CTT programmes on children's attendance at health centres

Country	Country income categories	Programme	Baseline (%)	Impact^α^	Significance^β^	Evaluation design^γ^
*Latin American and Caribbean countries*						
Chile	Middle	*Chile Solidario*	17.6	2.4(2.7)		RDD
Colombia	Middle	*Familias en Acción*	N/A	33.2(11.5)	^***^	DID
Ecuador	Middle	*Bono de Desarrollo Humano*	N/A	2.7(3.8)		RCT
Honduras	Middle	*Programa de Asignación Familiar*	44.0	20.2(4.7)	^***^	RCT
Jamaica	Middle	Program of Advancement through Health and Education	0.2	0.3(0.08)	^***^	RDD
Mexico	Middle	*Oportunidades*	0.2	0.03(0.02)		RCT
Nicaragua	Low	*Atención a Crises*	70.5	6.3(2.0)	^***^	RCT
Nicaragua	Low	*Red de Protección Social*	55.4	13.1(7.5)	^*^	RCT

*Source:* Fiszbein *et al*. (2009: 19–20).

*Notes:* N/A = not applicable or comparable with control baseline; α = the column for ‘impact’ reports the coefficient and standard error (in parentheses); the unit is percentage points except Jamaica where the unit is the number of visits to the health centre in the past six months, and Mexico, where the unit is the number of visits to the health centre in the last six months; β = significance levels: ^*^ < 0.05, ^**^ < 0.01, ^***^ < 0.001; γ = RCT defined in figure 2; DID = difference-in-differences examines treatment effect by comparing the treatment group both before and after treatment, and to a control group; RDD = regression discontinuity design elicits intervention causal effects by exploiting a given exogenous threshold determining assignment to treatment. By comparing observations lying closely on either side of the threshold, it is possible to estimate treatment effect in situations where randomization was not feasible.

**Table 2:** Impact of CTT programmes on children's attendance at school

Country	Country income categories	Programme	Baseline (%)	Impact^α^	Significance^β^	Evaluation design^γ^
*Latin American and Caribbean countries*						
Chile	Middle	*Chile Solidario*	60.7	7.5(3.0)	^***^	RDD
Colombia	Middle	*Familias en Acción*	63.2	5.6(1.8)	^***^	DID
Ecuador	Middle	*Bono de Desarrollo Humano*	75.2	10.3(4.8)	^**^	RCT
Honduras	Middle	*Programa de Asignación Familiar*	66.4	3.3(0.3)	^***^	RCT
Jamaica	Middle	Program of Advancement through Health and Education	18.0	0.5(0.2)	^**^	RDD
Mexico	Middle	*Oportunidades*	45.0	8.7(0.4)	^***^	RCT
Nicaragua	Low	*Atención a Crises*	90.5	6.6(0.9)	^***^	RCT
Nicaragua	Low	*Red de Protección Social*	72.0	12.8(4.3)	^***^	RCT
*Other countries*						
Bangladesh	Low	Female Secondary School Assistance Programme	44.1	12.0(5.1)	^**^	FE
Cambodia	Low	Japan Fund For Poverty Reduction	65.0	31.3(2.3)	^***^	DID
Cambodia	Low	Cambodia Education Sector Support Project	65.0	21.4(4.0)	^***^	DID
Pakistan	Middle	Punjab Education Sector Reform	29.0	11.1(3.8)	^***^	DID
Turkey	Middle	Social Risk Mitigation Project	87.9	−3.0	^*^	RDD

*Source:* Fiszbein *et al*. 2009: 17–18.

*Notes:* α = the column for ‘impact’ reports the coefficient and standard error (in parentheses); the unit is percentage points except Jamaica where the unit is days; β = significance levels: ^*^ < 0.05, ^**^ < 0.01, ^***^ < 0.001; γ = RCT defined in figure 2; DID = difference-in-differences examines treatment effect by comparing the treatment group both before and after treatment, and to a control group; FE = a fixed effects statistical model represents the observed quantities in terms of explanatory variables that are treated as if the quantities were non-random, often used in the analysis of panel data; RDD = regression discontinuity design elicits intervention causal effects by exploiting a given exogenous threshold determining assignment to treatment. By comparing observations lying closely on either side of the threshold, it is possible to estimate treatment effect in situations where randomization was not feasible.

**Table 3:** Impact of CTT programmes on food consumption

		Brazil	Colombia		Ecuador	Honduras		Nicaragua		
						
		2002	2002	2006	2005	2000	2002	2000	2001	2002
Daily per capita food consumption	Control	0.45	0.6	0.65	0.73	0.53	0.47	0.44	0.35	0.35
	Impact (%)	12^**^	N/A	6^**^	N/S	N/A	N/S		38^**^	31^**^
Food budget shares	Control (%)	60	74	56	54	71	72	73	69	68
	Impact (% points)	0.02^**^	N/A	0.04^**^	0.04^**^	N/A	N/S		0.04^**^	0.04^**^

*Source:* Fiszbein *et al*. 2009: 113.

*Notes*: significance levels: ^*^ < 0.05, ^**^ < 0.01, ^***^ < 0.001; N/A = not applicable or comparable with control baseline; N/S = no significant or discernable impact.

**Table 4:** Poverty measures for number of people (in millions) below $1.25 a day in 2005 purchasing power parities

Country	Headcount		Poverty gap^α^		Squared poverty gap^β^	
			
	Pre-transfer	Post-transfer	Pre-transfer	Post-transfer	Pre-transfer	Post-transfer
Brazil	0.2421	0.2369	0.0980	0.0901	0.0553	0.0471
Ecuador	0.2439	0.2242	0.0703	0.0607	0.0289	0.0235
Jamaica	0.2439	0.2329	0.0659	0.0602	0.0258	0.0224
Mexico	0.2406	0.2222	0.0847	0.0683	0.0422	0.0298

*Source:* Fiszbein *et al*. 2009: 110.

*Notes:* α = depth of poverty: this provides information regarding how far off households are from the poverty line; β = poverty severity: this takes into account not only the distance separating the poor from the poverty line (the poverty gap), but also the inequality among the poor. Thus, a higher weight is placed on those households who are further away from the poverty line.

## Welfare Transformed: ‘Workfare’ States in the Developed World

The neo-liberal foundations of the welfare state can be traced back to the postwar period, particularly in the USA. The 1990s, however, witnessed the full expression of this collective form of thinking. Mead and Beem (2005), for example, observe that major welfare reforms were undertaken in the advanced liberal democracies on ideological grounds, with an appeal to free-market political philosophy. Active labour market policies (ALMP), which have a tradition in the Nordic countries, were introduced into the liberal market economies with new duties (roles) and obligations (responsibilities) impressed on welfare benefit claimants. ‘Workfare’ (work-for-your-welfare) regulatory policies first appeared in the USA, thereafter work-based welfare reforms spread rapidly across the Organisation for Economic Co-operation and Development (OECD) countries (Nativel 2006). Notions of reciprocal obligation became firmly embedded in the various ‘welfare-to-work’ and ‘activation’ programmes. ‘Activation’ policy attempts to mobilize and enforce paid labour; work not only pays better than welfare, it is claimed, but also promotes well-being for individuals and nation states. Consequently, labour market conditionality now applies to most sections of the adult working age population across the developed world (Jacobsson and Noaksson 2010).

Currently, the primary obligation on social security recipients in most ‘workfare’ states is to actively look for paid employment, and in order to improve their chances, claimants must undergo training to develop new skills; they must engage with the employment services on offer for their own benefit. The original research that underpinned ‘welfare-to-work’ in the UK was encouraging, but certainly not convincing (Vincent *et al*. 1998).^3^ The new ‘workfare’ policy, however, was designed to solve a problem about the effects of passive, rights-and-eligibilities-based welfare that arose, it was argued, because too many citizens had been reduced to ‘welfare dependency’. Supporters of ‘activation’ argue the new duties impressed on welfare benefit claimants are perfectly justifiable as the social contribution now owed by citizens to society (Mead 1986). There is thus a strong ideological basis to ‘activation’ that sits squarely within the free-market neo-liberal paradigm. However, the research evidence supporting this great transformation of social policy has been the subject of much debate.

### Fragmentary evidence

Observers complain that research has not been driving welfare reform in the developed world in the way that it should (Haynes *et al*. 2012; Roberts *et al*. 2012). Aside from health, and possibly education (Oakley 2000), there appears a real lack of substantive investment in robust policy trials and systematic reviews. Research is only now beginning to piece together the fragmentary evidence relating to ‘activation’ in a systematic way (e.g. Kluve 2010; Marcia *et al*. 2012). The evidence from trials to date suggests only modest returns on certain activation programmes from unknown costs; notably, those that include job search activities accompanied by sanctions for non-compliance. However, the picture is as yet far from conclusive and findings may not be generalizable from one context to another (Dorsett *et al*. 2011; Hendra *et al*. 2011). Clearly ‘welfare-to-work’ programmes may have some obvious benefits as Lane *et al*. (2011) observe, providing tailored help and support to people who are looking for work, for instance. However, there may be structural concerns and policy limits. The present lack of employment opportunities is one (Dean 2012), child care costs are another (Davey and Hirsch 2011).

Until recently, ‘welfare-to-work’ evaluations have been rather limited in scope and design, particularly when considered against the large-scale trials in LMICs. Behavioural effects in the field of ‘activation’ are notoriously complex, difficult to observe and a challenge to monitor, particularly over time (Paz-Fuchs 2008). Often, there are indirect social and economic effects, unobserved consequences and population effects. Much of the evidence that has been generated seems patchy at best, meaning it is often inconclusive on key questions. For example, cost-benefit calculations are largely absent. ‘Welfare-to-work’ schemes in the UK have not delivered results, thus offering poor value to the British taxpayer (NAO 2012a). In some areas, ‘activation’ jobs are estimated to cost the public purse over £200,000 per new post (NAO 2012b). Subsequently, scandals make headlines in the British press and there have been high profile resignations amid allegations of fraud, excessive pay and bonuses for those charged with helping unemployed people back into paid work (Committee of Public Accounts 2012). Public confidence in the institutions may be shaken, but there may be popular support for many of the principles at stake here (discussed in the final section). Of course, politicians and policymakers are often keen to promote their policies and reforms. Thus, apparent and favourable impacts – improvements in employment rates or reductions in state expenditures, for instance – are seized upon to build public confidence and support. However, such information is usually uncontrolled knowledge. In other words, more often than not we know very little about the counterfactual outcome that would have been achieved had the programme not been in place.

## Arguments Concerning the ‘Use’ and ‘Misuse’ of Evidence and Evaluation

The review of global social policy, albeit brief, highlights a number of competing tensions and complex interrelated issues for thinking about the ‘use’ and ‘misuse’ of evidence and evaluation in policy-making that require further elaboration: these concern the nature of ‘evidence’; methodological and ethical issues; public attitudes and the nature of the policy process.

### Evidence

There is currently much debate over the nature and strength of the evidence emerging from CCTs. The evidence from LMICs, even for improvements on core programme objectives such as school attendance, is often unclear and context dependant, making it hard to generalize from one experiment to the next (Olken *et al*. 2012). Moreover, social interventions are often characterized by heterogeneity, delivered by different individuals operating in different social and geographical contexts. One implication of this heterogeneity is that average treatment and intervention effects, which are often the focus of attention, can be less useful than estimates of differential impacts across contexts (Plewis 2002). Increasingly, statistical models, and hierarchical models, are being used for estimating complex heterogeneity and different variances in the intervention and control groups; and this trend in modeling experimental and non-experimental social survey data is likely to continue (Byrne 2011).^4^

In the review we also find that evidence is weak or unproven in key areas of development, particularly for policies addressing child poverty (Yeates 2009) and child labour (Tabatabai 2009). Doubts are also expressed about the longer-term sustainability of programme benefits. Impacts tend to be evaluated in the short term and, as a consequence, little is known about longer-term effects. Observed benefits may be diluted over time. For example, evaluations tend to show the frailty of poverty reduction programmes (Lomelí 2009); jobs created by ‘activation’ programmes may not last (Kluve 2010). Structural issues extend far beyond individual agency and personal responsibility, as Wright (2012) observes. In times of high unemployment, for instance, there simply may not be enough jobs to go around (OECD 2012).

**Figure 5:**
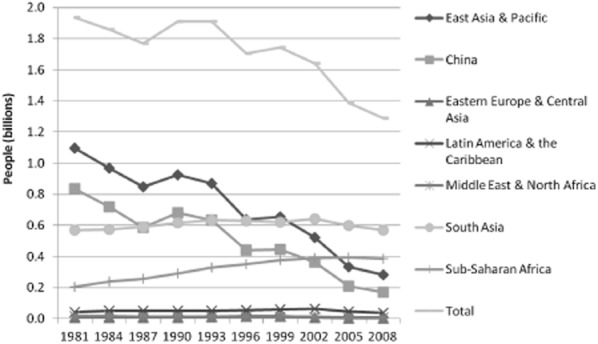
Estimates of chronic global poverty^α^ *Source:* Chen and Ravallion 2012: 5. *Note:* α = number of people living below $1.25 a day in 2005 purchasing power parities.

In addition, there are more fundamental, outright rejections of current neo-liberal policy prescriptions, which also appeal directly to the research evidence. ‘Washington Consensus’ policies, it is argued, are failing to make a real impact on chronic global poverty and deprivation (Townsend 2009; Anand *et al*. 2010). Extreme world poverty has fallen (Chen and Ravallion 2012), down from 1.94 billion people in 1981 to 1.29 billion people in 2008 (figure 5). However, nearly all of the poverty reduction during the period took place in China. Progress elsewhere, over nearly three decades, has been stubbornly slow, casting a long shadow of doubt over the ‘evidence-base’ supporting the present global policy response to poverty and deprivation (CSDH 2008). By contrast, a broad coalition of bodies under the United Nations recommends a global social protection floor to tackle the problem of chronic global poverty (ILO 2011), which would provide support to those not covered by adequate social security (four out of five people worldwide), for *unconditional* cash transfers are also effective and deliver many benefits for poor families. In Somalia, for example, the provision of cash grants to women not only improved food consumption levels and the use of health services, but also helped poor families to reduce their debts (Ali *et al*. 2005). Other countries, including India, are now beginning to trial and evaluate unconditional transfers; soon we may be able to assemble reliable comparative data to uncover the benefits of these schemes (Standing 2012).

### Evaluations

Social experiments in this field are often complex in design (Samson *et al*. 2010). Problems over administration and coordination occur, including poor targeting, and the costs involved in conducting an evaluation can be substantial (Lomelí 2008). Even in well designed trials there are a range of methodological issues to consider, such as bias in the results. For example, researchers have recently detected a possible ‘Hawthorne effect’ in some of the trial data from Latin America (de Brauw and Hoddinott 2011). This implies that some of the observed changes in behaviour during the course of a study may be related, at least in part, to the special conditions and monitoring systems in place during the period of investigation.

Despite the vast range of evaluations and RCTs, calculations of cost-benefit in this field are rare (St. Clair 2009). This makes it extremely difficult, if not impossible, to make clearly informed judgements about value for money, as Barrientos (2009) argues. The impact of conditionality is usually measured at the margins, by incremental gains. For example, secondary school enrolment rates for girls in Mexico's *Oportunidades* programme increased by 9 percentage points in two years, from a base of 67 per cent. Whether such modest gains are worth the costs is another matter, estimated at 2 per cent of the total social security programme budget in this case. Ideally, the overall (system) costs and benefits to individuals, families and society should be reflected in the evaluation. However, capturing the full range of information required is methodologically and technologically challenging, which itself comes at a cost. Conditionalities may, for example, impose non-trivial compliance costs on beneficiaries, which are not accounted for in their benefit levels. Conditionality imposes system costs as well as cost to individuals and families, although it is hard to know whether these costs are outweighed by the benefits, and if so, by how much. Including cost-benefit analysis in future evaluations would certainly help to inform the current debate. There is a compelling argument that *unconditional* transfers are cheaper, more efficient to administer and produce similar population benefits as previously discussed.

### Rights

Critics argue that social policy is being eroded by this newer neo-liberal brand advocated by the behaviour economists (e.g. Standing 2011). The issue at stake here is less about the ‘evidence-base’ – as we saw above, conditionality can be effective in changing behaviour in certain circumstances. The problem, according to some observers, is that conditionality undermines people's rights, citizenship and important principles governing social solidarity. CCTs are thus divisive, in that they create and sustain social divisions within society. Poor people are treated differently from other fellow citizens and this has major implications, for their basic human rights may be violated. Several international instruments affirm that every human being has the right to social security. For example, Article 22 of the 1948 UN Universal Declaration on Human Rights (reconfirmed in 1993 and 1996) states that ‘everyone, as a member of society, has the right to social security’ and Article 25 states that ‘everyone has the right to a standard of living adequate for their health and wellbeing’. If social security is only transferred on condition, then basic human rights are surely undermined. There are counter-arguments to this position, however, whereby exponents claim that conditionality represents an instrument of policy that, in practice, confronts the denial of basic human rights by ensuring that welfare reaches families most in need (see Lomelí 2008).

### Power

Critics claim that conditionality in public policy reinforces entrenched inequalities within and between societies, and thus preserves existing social structures and power relations (e.g. Mcdonald and Marston 2005; Veit-Wilson 2009). This discourse attempts to move us on from fairly constrained debates about the ‘evidence-base’ of policy to much more basic concerns regarding the legitimacy of current policy programmes, and the ongoing power struggles between the dominant elites and the dominated poor. Thus, many policymakers seem to believe that poor people must be threatened with less money to incentivize them to follow predefined behaviour patterns, but this form of practice constitutes poor people as subjects engaged in a power struggle for resources, recognition and respect. Subsequently, we need to be much more critical about how ‘social problems’ are defined, as Bacchi (2009) argues, and the ways in which research ‘evidence’ is represented for policy-making.

### Ethics

RCTs (as a method of evaluation) can raise profound ethical issues. RCTs involving conditionality (i.e. policy intervention) raise even more concerns because they antagonize people in two distinct camps. Critics of RCTs, for example, argue that it is unfair to experiment with the lives of poor vulnerable and disadvantaged people (Elizabeth and Larner 2009). Experiments with social security and assistance – by their very nature – often target the most vulnerable sections of society. Therefore, critics claim that conditionality undermines social inclusion or, worse still, reinforces existing prejudices in the community. Unemployed people are often stigmatized in the British media, for instance (e.g. Walters 2012). For this reason, the Roman Catholic Church in Australia principally opposes the inclusion of Indigenous families in Australia's new income management scheme (Quinlan 2010). In this programme, a range of benefits including unemployment, disability and single parenting payments, are granted on condition that recipients do not spend their money on goods such as alcohol, tobacco, pornography or gambling products (FaHCSIA 2010). The programme involves elaborate systems for monitoring compliance and controlling behaviour which, in some senses, seeks to attract a degree of social acceptability. Ethically, however, the scheme has been the subject of much controversy. In many ways, the key question turns on what counts as ‘evidence’, and to whom – and the extent to which – we can include notions of ‘social justice’ and ‘human rights’ in the framework of RCTs and evaluation. Ultimately, the design of welfare policy invokes our values and the values and attitudes of our fellow citizens, which may conflict and contrast in sophisticated ways.

### Public attitudes

Whilst implementing welfare reforms with little or no clear evidence of their effectiveness may offend those interested in evidence-based policy-making, and is itself unethical (Davies *et al*. 2000), we can recognize that ‘evidence’ comes in a variety of forms, and make room for broad conceptions of ‘evidence’ and ever-shifting social attitudes. Public attitudes and perceptions have an important place in the policy-making process. Standing (2008), for example, sees the CCT initiative in developing countries as a political device to legitimize cash transfers with middle-class voters and international agencies. In the developed world, Taylor-Gooby (2011) argues that welfare reform has become politically easier and more acceptable, thanks to recent changes in public attitudes. Analyzing the UK context, he observes hardening attitudes towards welfare, thus allowing the Coalition government to drive through radical reforms in an effort to cut public spending. Over half of the British public now think that welfare benefits are too high, with disincentive effects for work (Park *et al*. 2011). Reciprocity may help to legitimize the welfare state function according to some, but equally the shift can be seen to undermine the principles of social policy as social solidarity. A more mixed picture emerges across the developed world, where in the Nordic and Southern European countries, for instance, the majority of people appear to support the unconditional model of welfare, whereas in the Anglo-Saxon nations it is the conditional model that is currently in favour (Jæger 2011).

The issues involved here are notoriously complex, however, and can invoke a ‘policy sciences of tyranny’ (Dryzek 1989). For example, the British general public may support elements of conditionality in welfare policy, but large sections of the population who find themselves ‘on condition’ may object to the policy. Nearly two-thirds of lone parents in the UK, for instance, dislike or reject the conditions imposed upon them by the new ‘welfare-to-work’ regulatory regime: they would prefer to look after their own children themselves rather than be forced into work and have to pay for child care services (Rafferty and Wiggan 2011). Hence, the evidence seems to suggest their needs and interests, and possibly those of their children, are not respected under the new policy. Income management trials in Australia revealed strong support from welfare beneficiaries (FaHCSIA 2010): some 70–80 per cent of participants reported positive benefits for themselves and their families; six in ten participants said the scheme had helped in improving the lives of their children and just under half claimed it had done so significantly. These findings were used by policymakers to help justify the national policy, which was rolled out across Australia in 2012. Social surveys are not always value-free instruments, of course (Goerres and Prinzen 2012), but even if conditionality in policy improves levels of living for some families in ways that they approve of, it is highly questionable whether these same families would accept or consider appropriate the sanctions imposed when conditions are not met – particularly if child well-being was at stake.

### The policy process

Conditionality in behavioural economics is often presented as a ‘model’ to be tested within the positivist tradition of social science. However, the implicit assumption of a linear relationship between research evidence and policy is not easily supported (see Parsons 2002), especially where political ideology is inextricably tied up in the policy process, as Wiggan (2012) discovered. Work by Stubbs (2009) reveals how programme goals in this field are socially constructed. Drawing on CCT experiences in Eastern Europe, Stubbs observes how policymakers negotiate the aims and objectives of their programme, adapting trial evidence from one context (Latin America) to another (Europe). The historical, cultural and institutional connections here are tenuous at best, he argues, and families may suffer as a result of the policy translation process, as welfare is reformed. From this perspective, there is no real ‘gold standard’ approach; judgements about evidence and policy are required on a case-by-case basis (Cartwright 2007). Often, policy conclusions cannot simply be exported from one population or context to another.

## Discussion

The world of public policy has been radically transformed in recent times, but here we have a tale of two worlds. The great transformation of welfare within the developed world is arguably a result of political thinking and ideology; today the research effort is largely focused on trying to embed complex ‘workfare’ systems, in an effort to make them work more effectively. In the global south, policy appears to be more driven by the evidence-based policy agenda for human development. Ultimately, this seems to be about ensuring scare funds are spent wisely to meet the MDGs, which on many levels is unobjectionable. Strengthening the evidence-base for policy decisions is therefore seen as a positive move. However, the whole ‘evidence-based’ approach to policy itself can be seen to be ideologically driven, as Packwood (2002) argues. In other words, CCT policies arguably support particular beliefs and values compatible with the dominant neo-liberal paradigm – promoted by the ‘Washington Consensus’ – that increasingly defines how people and society should function in the 21st century (Draibe and Riesco 2009).

So far, governments in the developed world have largely resisted the temptation to impose the sorts of behavioural conditions on welfare recipients seen in LMICs, although this may be changing. In Australia, for example, child benefit started to be conditioned on compliance with national healthcare requirements, while the new income management programme goes much further with behavioural conditioning. In the UK, the government is considering stronger ‘workfare’ measures for the long-term unemployed, who otherwise risk losing all their benefits. Social policy, it would seem, is becoming increasingly interventionist, and conditioned (e.g. Thaler and Sunsyein 2009; Behavioural Insights Team 2011). Governments are turning to behavioural economics and ‘libertarian paternalism’ to tackle complex ‘social problems’, often resulting from current lifestyles of apparent ‘excess’ (Le Grand 2008; HM Government 2010). In so doing, new systems of governance for monitoring and controlling our behaviour, designed to enhance social justice and human well-being, are firmly established, as the behavioural psychologist B. F. Skinner famously anticipated (Skinner 1948).

So what lessons might we draw from this review? A basic question within social policy, which is rarely addressed directly or well (the work by Saunders 2002, is exceptional in this regard), is to ask whether the ends of welfare justify their means. This review offers three different, and partly conflicting, answers to this question:

- The ends justify the means. There is no shortage of good solid evidence from robust evaluations and systematic-reviews in LMICs that show significant benefits and great strides in social progress and human flourishing (e.g. tables 1–4). Developed countries could learn much from social experimentation with social policies that include robust evaluations.
- The ends do not justify the means. The behavioural approach to tackling global social problems such as chronic poverty is failing (e.g. figure 5). Evaluations are often flimsy and evidence is overstated. At best, we find marginal gains for untold costs (e.g. tables 1–4).
- Maybe the ends justify the means. However, there is much uncertainty, results are often inclusive or conflicting and there are plenty of ‘information gaps’. We simply do not know at present whether the ends justify the means. The lack of cost-benefit data and trials of alternative models (e.g. unconditional transfers) are major impediments in this field.

All three positions carry great weight in the social and policy sciences at present. Ultimately, then, the ‘use’ and ‘misuse’ of evidence and evaluation appear to lie in the eye of the beholder. Those advocating neo-liberal forms of policy, particularly with conditionality applied to social security or assistance, argue the ‘use’ of evidence and evaluation can be justified both in principle and practice. Those who argue for ‘misuse’ flatly reject this. The issues discussed here cannot easily be solved by appeals to the ‘evidence’ alone, at least how things stand, and while better evaluations may help, they are sure to stir and provoke our values and principles still further.

## Notes

1 To be included in a systematic review or meta-analysis, a study must usually meet strict research quality criteria relating to research design, often this usually means a randomization or a well designed before-and-after control study. A clear measure of at least one outcome is also required.

2 The International Development Coordinating Group website of the Campbell Collaboration has further information on systematic reviews completed and in-progress, http://www.campbellcollaboration.org/international_development/index.php (accessed 14 March 2013).

3 This study published in 1998 appears to be the first RCT in the field of social security in Britain.

4 A statistical model, underpinned by mathematical equations, is a formalization of relationships between variables. The model is statistical as the variables are not deterministically related but stochastically (best fit) related.
